# Mind the gap—national pesticide monitoring data needs for invertebrate effects assessments in English rivers

**DOI:** 10.1093/etojnl/vgae087

**Published:** 2025-01-06

**Authors:** Imogen P Poyntz-Wright, Charles R Tyler

**Affiliations:** Biosciences, Geoffrey Pope Building, University of Exeter, Exeter, United Kingdom; Biosciences, Geoffrey Pope Building, University of Exeter, Exeter, United Kingdom

**Keywords:** pesticides, monitoring, rivers, risk assessment

## Abstract

Pesticides are an integral part of agriculture in arable and pastoral farming and in animal and pet care, but they have been shown to have detrimental impacts on biodiversity, including of freshwater systems. The United Kingdom (UK) has the 7th highest pesticide usage per area of arable land across 30 European and African countries assessed between the years 2000 and 2012 and thus an associated higher likelihood for impacts on riverine biodiversity. In our analysis of the UK’s 24-year national chemical monitoring program (WIMS database; years 2000 to 2023), we show that of the nine pesticides that pose the greatest likely threat to UK freshwater invertebrates based on concentrations measured in British rivers exceeding the lowest effect concentrations (ECs) in laboratory-based toxicity tests, seven pesticides have exceeded the ECs across England between the years 2000 and 2023. The Anglian and Midland regions of England that have the highest regional arable pesticide use recorded the greatest number of pesticides exceeding the ECs for aquatic invertebrates. However, this finding may also be influenced by the more limited sampling/monitoring bias across England, and greater sampling of southern and northwest rivers is needed to better establish the potential impact of pesticides on riverine invertebrate communities in those regions.

## Introduction

Globally, pesticides are an integral part of pest management in agriculture and in livestock and pet (animal) care; however, their use, especially through arable farming practices, has been associated with detrimental impacts on biodiversity, including on riverine invertebrate communities (Dijk et al., 2013; Schäfer et al., 2007; Poyntz-Wright et al., 2024) that play fundamental roles in aquatic ecosystem function (Wallace & Webster, 1996). Understanding chronic exposure impacts of pesticides on riverine invertebrate populations requires comprehensive monitoring data, which is both costly and time consuming, and therefore needs to be effectively targeted for maximizing support of environmental projection.

Across Europe, the United Kingdom (UK) uses some of the greatest quantities of pesticides per hectare of agricultural land (Sharma et al., 2019). As a consequence, UK riverine invertebrates are likely to be more vulnerable to arable pesticide exposure. To enable assessments on pesticide exposure effects on aquatic biota, the UK Environment Agency has one of the most comprehensive national chemical monitoring programs globally (WIMS database; https://environment.data.gov.uk/water-quality/view/landing). In this program, the pesticides monitored are guided by statutory requirements to monitor those listed in certain directives, including the Water Framework Directive, ensuring compliance (Environment Agency, 2013a). Further nonstatutory sampling occurs to investigate known or predicted local problems and pollution incidents (Environment Agency, 2013a).

Here, we analyzed the UK’s Environment Agency chemical monitoring data collected over a 24-year period (years 2000 to 2023) to assess for exposure of pesticides and to identify the pesticides of most concern for riverine invertebrates regionally across England. We show a strong bias in pesticide monitoring in the Anglian and Midland regions of England and lack of monitoring in the Northwest, Southeast, Southwest, and Thames regions and thus a gap for understanding the UK’s national picture for effects of pesticides on riverine invertebrate biota.

## Methods

Utilizing the WIMS Environment Agency database for years 2000 to 2023 (https://environment.data.gov.uk/water-quality/view/landing), we first calculated the number of samples collected for pesticide residue analysis from riverine sites across England and the number of samples analyzed annually for each study region (Anglian, Midlands, Southwest, Southeast, Thames, Northeast, and Northwest; see online supplementary material Figure S1).

We then identified pesticides of concern to British riverine invertebrates based on those in British rivers exceeding the lowest concentration that causes a population-relevant effect (reproduction, growth, development and mortality) from laboratory studies on British freshwater invertebrates. The concentrations of pesticides in British rivers were defined based on an analysis of the historical concentrations reported in the literature. We used Web of Science (query—TS=(*pesticide concentration* OR *pesticide level*), AND TS=(Riverine OR “surface water”), AND TS=(British OR Britain OR England OR English OR UK; returned 36 papers)) to identify articles that include previously monitored pesticides in British rivers. Google Scholar was used as an additional source to identify any missed papers (query- “pesticide concentrations” OR “pesticide levels”) AND (British OR UK OR England OR English OR Wales) AND rivers AND surface water; see online supplementary material Table S2 for the articles that monitored each compound and the regions where each compound was monitored). For each pesticide, we identified the lowest concentration that caused a population relevant effect on a British riverine invertebrate. The lowest effect concentrations for each pesticide were established using the ECOTOX database (https://cfpub.epa.gov/ecotox/), in which data are reported as either lowest observed effect concentration, median lethal concentration (LC50), half-maximal effect concentration (EC50), EC20, EC25, or LC01, depending in part on the endpoint measured. To help avoid confusion across all these different measures, in this article, the lowest concentration that caused a population relevant effect for each pesticide is simply referred to the effect concentration or EC. The National Biodiversity Atlas database was used for validation of British taxa (https://nbnatlas.org/; see online supplementary material Table S2 for information regarding the species sensitivity, test duration for each population relevant endpoint [growth, development, reproduction and mortality] per pesticide). Using hazardous concentration for 5% of species (HC5) and species sensitivity distributions (SSD) to determine the risk of pesticides to invertebrate communities was considered for the work in this study, as used by Schroer et al. (2004) for studying the impacts of λ-Cyhalothrin. However, there were not sufficient data available on most pesticides across the range of British invertebrate species to do so effectively at this time.

Of the pesticides measured in British rivers, nine had concentrations exceeding ECs for British freshwater invertebrates and were monitored in the Environment Agency’s WIMS database. The nine pesticides were organophosphates (chlorpyrifos-ethyl, diazinon, fenitrothion, parathion, and malathion), pyrethroids (cypermethrin and deltamethrin), organotin (tributyltin and organochlorine) and lindane. Chlorpyrifos and cypermethrin were among the top 50 pesticide active substances most extensively used on arable crops and amenities in Britain between years 2000 and 2022 (Food and Environment Research Agency, 2000, 2006, 2008, 2010, 2012, 2014, 2015, 2016a, 2016b, 2018, 2020a, 2020b, 2022). Subsequently, using the WIMS database, we calculated the average (mean) concentrations of these nine individual pesticides of concern to British riverine invertebrates per year for all riverine sites monitored for each geographical region. We used mean values as a way to capture the full range of pesticide concentrations measured, but we acknowledge that mean values can be influenced by “outlier” data points, albeit these outliers may represent true values given the episodic nature of pesticides run off into rivers. We have thus provided graphs in the supplementary materials (S4 to S12) that show all the individual data measurements for each pesticide for each region.

Limitations on the availability of temporal pesticide monitoring data for the Thames region (data available for a single year only) and the Northeast, Southeast, and Southwest (available from years 2015, 2018, and 2019 onwards, respectively) prevented a statistical analyses to assess for differences in pesticide concentrations monitored between the regions (See online supplementary material Figure S2).

## Results

### Pesticide sampling across England

**Figure 1.**
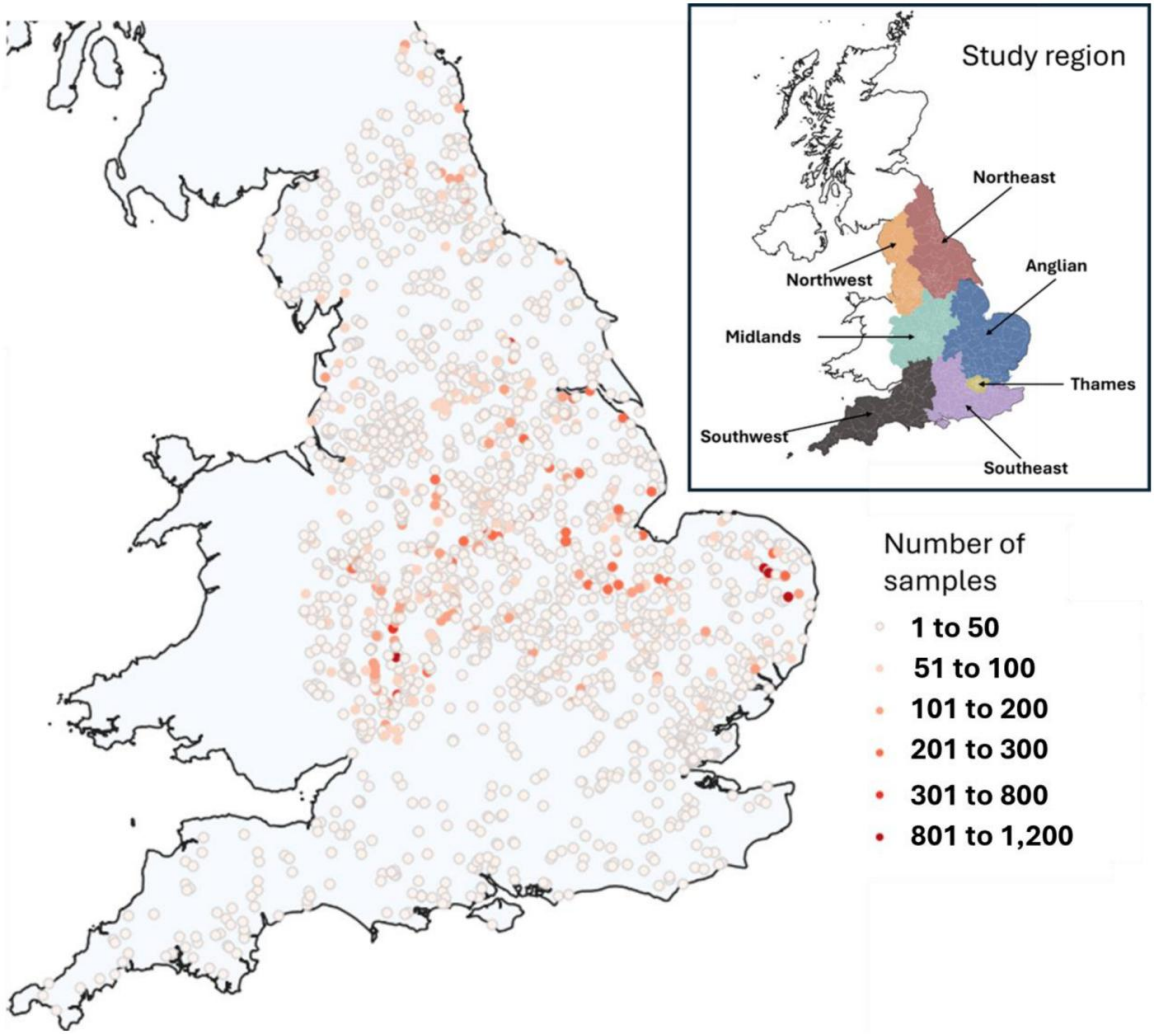
Maps showing sampling sites and intensity of samplings for pesticides in England for years 2000 to 2023. The key provided illustrates the number of pesticide samples collected over the entire 24-year monitoring period. Each dot represents the location of a riverine sampling site.

Between 2000 and 2023, fewer than 50 samples were collected for pesticide residue analysis from the majority of riverine monitoring sites (see Figure 1) and no riverine sites in the Southwest, Southeast, and Thames regions had more than 50 samples collected in total for pesticide residue analysis. In contrast, the Midland and Anglian regions of England received more comprehensive sampling efforts, with samples collected every year over the 24-year period and with some riverine sites having more than 800 samples collected for pesticide residue analysis (See online supplementary material Figure S4). For the Northwest, Southwest, Southeast, and Thames regions, riverine samples for pesticide analysis were collected from 2015 onwards only and for the Thames region for the year 2020 only.

### Concentrations of pesticides monitored in English rivers

Average (mean) pesticide concentrations varied considerably across the different regions between the years 2000 and 2023. For the nine pesticides assessed that exceeded the ECs for population relevant endpoints (reproduction, growth, development, and mortality) for freshwater invertebrates, the highest mean concentrations were observed in the Anglian, Midland, and Northwest regions. The Anglian region had the highest mean concentrations for cypermethrin, fenitrothion, lindane, malathion, parathion, and tributyltin; the Midland region for chlorpyrifos-ethyl and deltamethrin, and the Northwest region for diazinon. Over the monitoring period, the pesticides diazinon and tributyltin were consistently at higher-than-average concentrations in the Northwest and Anglian regions, respectively. The concentrations of all these pesticides were relatively low in the Thames, Southwest, Southeast, and Northeast regions.

### Potential risk of pesticides to riverine invertebrates in English rivers

**Figure 2.**
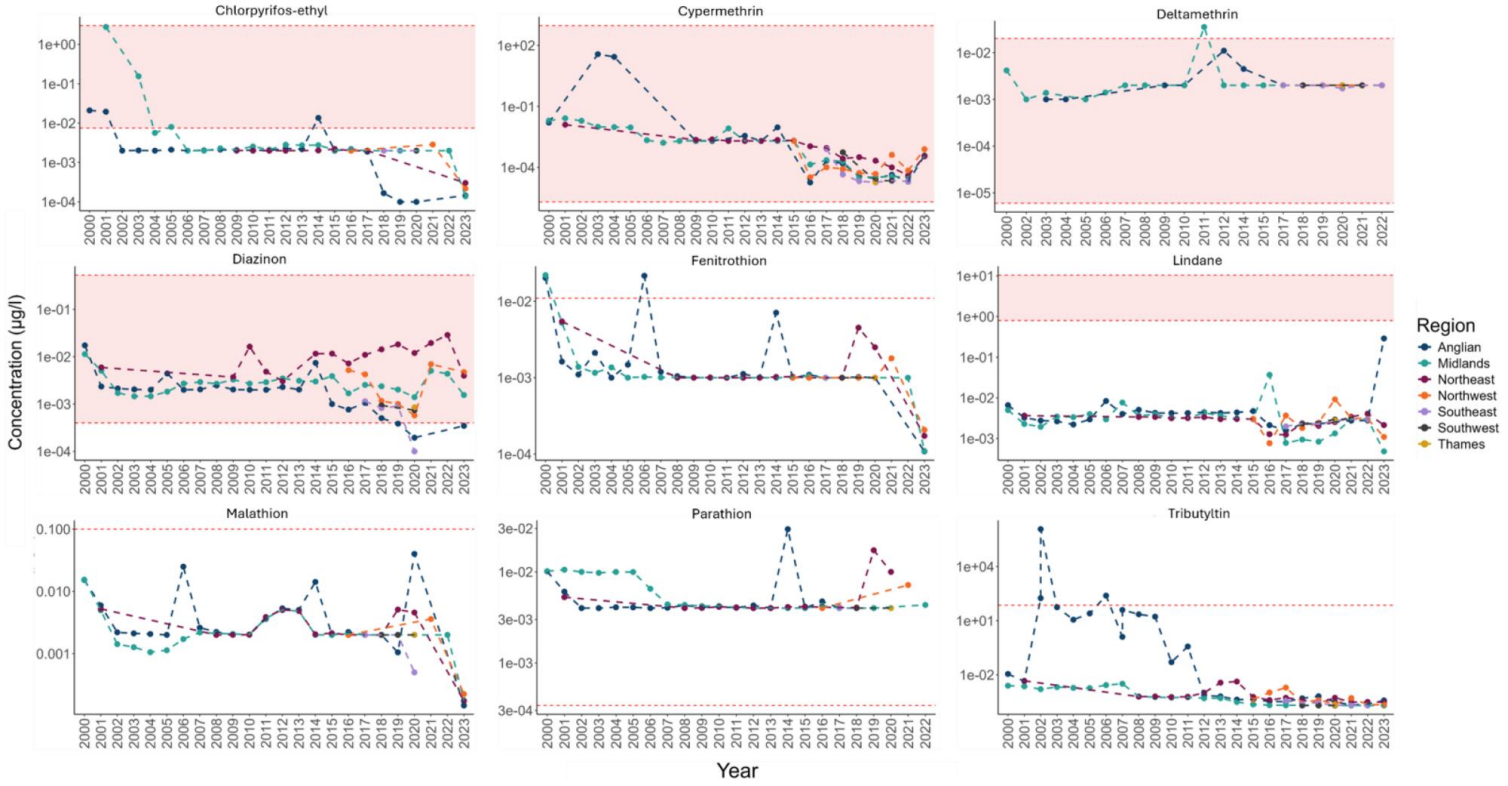
Mean concentration of nine pesticides recorded exceeding the lowest effect concentrations (ECs) for population relevant endpoints for freshwater invertebrates across riverine sites in the different geographical regions of England between the years 2000 and 2023. Shaded vertical (red) bands/lines indicate the ECs for a population relevant endpoints (See online supplementary material Table S2). Y-axis uses a log base 10 scale.

The average (mean) measured riverine concentrations for four of the above nine pesticides exceeded the EC for a population-relevant endpoint across all regions (Figure 2). In the Anglian region, the mean measured riverine concentrations for a further three pesticides exceeded the ECs. The frequency for exceedances of the ECs differed for different pesticides and regions (Table 1), ranging between 0.01% and 100% of the riverine samples analyzed.

**Table 1.** Exceedances of lowest effect concentrations for nine pesticides for British freshwater invertebrates in English rivers for different geographical regions, using the UK National Monitoring Programme (WIMS) database from 2000 to 2023.^a^

Chemical	Endpoint	Percentage of samples which have exceeded endpoints per region						
		Anglian	Midland	Northeast	Northwest	Southeast	Southwest	Thames
**Chlorpyrifos-ethyl**	Mortality (0.02 µg/L^6^)	1% (*n* = 32)	1% (*n* = 21)	0.2% (*n* = 1)				
	Reproduction (0.021 µg/L^5^)	0.3% (*n* = 11)	0.9% (*n* = 19)					
	Growth (3 µg/L^1^)		0.09% (n = 2)					
	Development (7.5E-03 µg/L^1^)	1% (*n* = 42)	3% (*n* = 62)	0.2% (*n* = 1)	2% (*n* = 3)			
	Mean concentration of samples (µg/L):	0.00365	0.0116	0.00186	0.00110		0.002	0.002
**Cypermethrin**	Mortality (0.002 µg/L^1^)	38% (*n* = 883)	50% (*n* = 1,387)	44% (*n* = 1,148)	7% (*n* = 108)	4% (*n* = 18)	2% (*n* = 3)	
	Reproduction (2×10^–6 ^µg/L^1^)	100% (*n* = 2,357)	100% (*n* = 2,792)	100% (*n* = 2,614)	100% (*n* = 1,475)	100% (*n* = 475)	100% (*n* = 170)	100% (*n* = 52)
	Growth (950 µg/L^1^)	0.4% (*n* = 1)						
	Mean concentration of samples (µg/L):	2.09	0.00212	0.00127	0.000199	0.000102	0.0000565	0.0000188
**Deltamethrin**	Mortality (6×10^–6 ^µg/L^1^)	100% (*n* = 594)	100% (*n* = 301)	100% (*n* = 458)	100% (*n* = 685)	100% (*n* = 199)	100% (*n* = 137)	100% (*n* = 9)
	Reproduction (5.4×10^–3 ^µg/L^5^)	1% (*n* = 6)	1% (*n* = 3)					
	Growth (0.005 µg/L^1^)	1% (*n* = 6)	1.3% (*n* = 4)					
	Development (0.02 µg/L^1^)	0.6% (*n* = 4)	0.7% (*n* = 2)					
	Mean concentration of samples (µg/L):	0.00203	0.00260	0.002	0.002	0.00199	0.002	0.002
**Diazinon**	Mortality (3.5 × 10^–4 ^µg/L^2^)	92% (*n* = 2,784)	99% (*n* = 9,105)	90% (*n* = 1,940)	86% (*n* = 510)	60% (*n* = 52)	59% (*n* = 13)	81% (*n* = 9)
	Reproduction (2 ×10^–4 ^µg/L^3^)	94% (*n* = 2,840)	99% (*n* = 9,142)	93% (*n* = 2,007)	92% (*n* = 551)	70% (*n* = 61)	72% (*n* = 16)	90% (*n* = 10)
	Growth (0.53 µg/L^3^)	0.3% (*n* = 2)	0.01% (*n* = 1)	0.5% (*n* = 1)				
	Mean concentration of samples (µg/L):	0.00246	0.00345	0.0107	0.00448	0.000955	0.00091	0.000853
**Fenitrothion**	Reproduction (0.011 µg/L^1^)	4% (*n* = 164)	13% (*n* = 1,051)	0.04% (*n* = 1)				
	Growth (0.011 µg/L^1^)	4% (*n* = 164)	13% (*n* = 1,051)	0.04% (*n* = 1)				
	Mean concentration of samples (µg/L):	0.00299	0.00375	0.00114	0.000838	0.001	0.001	0.001
**Parathion**	Mortality (3.1 × 10^–4 ^µg/L^2^)	100% (*n* = 2,695)	100% (*n *= 6,551)	100% (*n* = 2,155)	100% (*n* = 52)	100% (*n* = 18)	100% (*n* = 6)	100% (*n* = 9)
	Mean concentration of samples (µg/L):	0.00742	0.00713	0.00427	0.007	0.004	0.004	0.004
**Tributyltin**	Mortality (72 µg/L^2^)	0.9% (*n* = 41)						
	Mean concentration of samples (µg/L):	279	0.000739	0.00130	0.000733	0.000261	0.0002	0.000212
**Lindane**	Mortality (0.8 µg/L^2^)	0.05% (*n* = 3)	0.05% (*n* = 3)					
	Reproduction (10.5 µg/L^1^)	0.01% (*n* = 1)						
	Growth (6.11 µg/L^1^)	0.01% (*n* = 1)						
	Mean concentration of samples (µg/L):	0.00932	0.00448	0.00257	0.00260	0.00254	0.00284	0.00277
**Malathion**	Mortality (0.25 µg/L^1^)	0.1% (*n* = 4)						
	Reproduction (0.1 µg/L^1^)	0.2% (*n* = 6)	0.04% (*n* = 3)					
	Growth (0.1 µg/L^1^)	0.2% (*n* = 6)	0.04% (*n* = 3)					
	Mean concentration of samples (µg/L):	0.00505	0.00366	0.00258	0.00142	0.00190	0.002	0.002

a Lowest observed effect concentration (LOEC) = ^1^, LC50 (lethal concentration of 50%) = ^2^, EC50 (effect concentration of 50%) = ^3^, EC20 (effect concentration of 20%) = ^4^, EC25 (effect concentration of 25%) = ^5^, LC01 (lethal concentration of 1%) = ^6^.

## Discussion

For nine pesticides, concentrations at riverine sites across regions monitored in England (Anglian, Midlands, Southwest, Southeast, Thames, Northeast, and Northwest) exceeded the ECs for population-relevant effect measures for British riverine invertebrate species, with the potential for harmful effects to those populations. The Anglian and Midlands regions (the regions most intensely monitored) most frequently saw exceedance of the ECs for seven of the nine pesticides highlighted above, indicating a greater risk for harm to riverine invertebrate communities in these regions (Table 1 and Figure 2). This finding is supported by recent studies of those regions assessing for associations between the presence of those pesticides and bioindicator taxa (species at risk) and through modeling studies (Poyntz-Wright et al., 2023, 2024). There is, however, greater uncertainty for the pesticide assessment data for the Southwest, Southeast, Thames, Northeast, and Northwest where there has been more limited regional sampling. This is especially concerning regarding the national picture, which suggests river quality in agriculturally intense catchments likely remains worse now than before the 1960s, and that pesticide pressure has increased particularly for pyrethroids triazole fungicides and sulfonylurea herbicides (Whelan et al., 2022).

The analyses conducted shows regional and temporal differences in the types of pesticide pressures for rivers across the different regions. Diazinon, for example, a pesticide used to control insects in soil for ornamental plants and on fruit and vegetables and that is extremely toxic to invertebrates generally (See online supplementary material Table S2; *Daphnia magna*), was found consistently at higher concentrations in the Northwest region over the years 2001 to 2023 when comparted with other regions in England. Similarly, the presence of tributyltin, a chemical used in antifouling paints, slime control, disinfectants, and wood preservatives, was consistently found at high concentrations in the Anglian region between 2002 and 2011. However, since 2007, diazinon has been restricted to veterinary applications and is no longer authorized for agricultural use, whereas tributyltin was banned from use in paints in 1987 (Dowson et al., 1993; GOV.UK, 2007).

The focus of environmental monitoring for pesticides in the Anglian and Midlands regions of England is justified given the high levels of arable (crop) farming that occur in these regions (Smith et al., 2018) and thus likely associated higher risk for pesticide exposure in the riverine systems in these regions. However, considerable amounts of pesticides are also used in arable farming across other regions of England, especially in the Southeast and Thames regions (Smith et al., 2018). In addition, pesticide monitoring data are available only since 2018 and no data are available for 2023 for these regions, identifying a further data gap. It is also now well recognized that various pesticides used in veterinary medicines for livestock care, including anti-helminthics such as ivermectin and doramectin (Environment Agency, 2006), and in pet care, for example, imidacloprid and fipronil, used for flea treatments (Perkins et al., 2021) and are highly toxic to invertebrates, are entering waterways, thus posing a potential threat riverine invertebrates, but these compounds have rarely been detected/monitored as part of the UK National Monitoring Programme (WIMS). Moreover, many pesticides of the top 15 most used in Britain from 2000 to 2022 (Food and Environment Research Agency, 2000, 2006, 2008, 2010, 2012, 2014, 2015, 2016a, 2016b, 2018, 2020a, 2020b, 2022), including mancozeb, perosulfocarb, prothioonazole, folpet, tebuconazole, flufenacet, propamocarb hydrochloride, metamitron, alconifen, spiroxamine, epoxiconazole, and boscalid, have not been monitored in UK rivers as part of the National Monitoring Programme.

The UK government’s 25-year environment plan (Department for Environment, Food & Rural Affairs, 2022) relating to chemical pollution explicitly states it will reduce chemical pollution (including agricultural pollution) in British waters and improve riverine biodiversity, particularly as applied to the use of pesticides for crop protection. The UK’s revised National Action Plan for pesticides (Environment Agency, 2013b) also sets out to minimize the risk and impact of pesticides to the environment while ensuring pest and pesticide resistance is managed. Achieving such objectives, however, will require deeper, regionally explicit information on the levels of pesticides in the riverine environment. In addition, inclusion of some of the pesticides used widely for parasite control in livestock and pet animal care that are known to be highly toxic to invertebrates is much needed. These chemical data need to be collected at the same sites as the monitoring sites of freshwater biota populations to enable accurate calculations on the risk pesticides pose to riverine invertebrate populations for chronic exposures. Greater monitoring is especially required in Southern regions of England to enable any effective evaluation of pesticide risk to riverine invertebrate populations for those regions.

## Supplementary Material

vgae087_Supplementary_Data

## Data Availability

Data, associated metadata, and calculation tools are available from the corresponding author (ipp203@exeter.ac.uk or c.r.tyler@ex.ac.uk).
