# Supplementary material for: Mind the gap—national pesticide monitoring data needs for invertebrate effects assessments in English rivers
Source: Environ Toxicol Chem. 2025 Jan 6;44(3):637–42. doi: 10.1093/etojnl/vgae087 (PMC11864203; doi:10.1093/etojnl/vgae087)
Supplement: vgae087_Supplementary_Data [file vgae087_supplementary_data.zip › vgae087_Supplementary_Data/Supplementary Information 03_12_24.docx]

Supplementary Information

Mind the Gap- Nationale Pesticide Monitoring Data Needs for Invertebrate Effects Assessments in English Rivers

Pages - 21

Tables - 2

Figures - 12

**Table S1: Summary of the number and percentage of pesticides per class identified in English rivers between 2000 and 2023**

| **Class of pesticide** | **Number of chemicals classified** | **Percentage of chemicals classified** |
| --- | --- | --- |
| Insecticide | 103 | 50.24 |
| Herbicide | 74 | 36.10 |
| Fungicide | 34 | 16.59 |
| Acaricide | 10 | 4.88 |
| Biocide | 5 | 2.44 |
| Nematicide | 4 | 1.95 |
| Molluscicide | 2 | 0.98 |
| Plant Growth Regulator | 1 | 0.49 |
| Avicide | 1 | 0.49 |
| Rodenticide | 1 | 0.49 |
| Miticide | 1 | 0.49 |

*205 pesticides were identified, some pesticides are used across various classes. The majority of pesticides (>80%) are used as insecticides and/or herbicides.

**Table S2: Pesticides where the lowest effect concentrations for UK freshwater invertebrates derived from laboratory-based studies are exceeded in British rivers**

| **Chemical** | **Field concentrations (µg/l)** | **Number of samples** | **Region** | **References** | **Lowest effect concentrations which exceed field concentrations (µg/l)** | **Number of riverine samples in the Environment Agency’s WIMS database (2000 – 2023)** | **Percentage of riverine samples from the WIMS database that exceed lowest effect concentrations** |
| --- | --- | --- | --- | --- | --- | --- | --- |
| 2-4-D | <0.05 – 2.1 | 1012 | England: Southeast, Anglian | (Neal *et al.*, 2000) (Croll, 1991) |  | 16766 |  |
| Acetamiprid | 0 – 0.192 | 369 | Scotland  Northern Ireland  Wales  England: Northeast, Northwest, Southeast, Thames, Midlands, Anglian | (Buglife, 2017) (Egli *et al.*, 2023) |  |  |  |
| Atrazine | 0.001 – 7.1 | 1961 | England: Southeast, Northeast,  Southwest,  Anglian,  Thames | (Neal *et al.*, 2000) (Long *et al.*, 1998) (Casado, Santillo and Johnston, 2018) (Egli *et al.*, 2023) (Spurgeon *et al.*, 2021) (Croll, 1991) |  | 16357 |  |
| Azoxystrobin | 0.0007 - 0.76 | 2435 | England: Southwest,  Thames | (Spurgeon *et al.*, 2021)  (Casado, Santillo and Johnston, 2018)  (Egli *et al.*, 2023) |  | 2054 |  |
| Bentazone | 0.0029 - 51 | 5998 | England: Anglian, Southwest, Midlands, Southeast, Thames, Northeast, Northwest  Wales | (Environment Agency, 2005)  (Spurgeon *et al.*, 2021)  (Casado, Santillo and Johnston, 2018)  (Comber, Mistry and Sturdy, 2012) |  | 8539 |  |
| Boscalid | 0.0031 - 100 | 2820 | England: Southwest | (Spurgeon *et al.*, 2021)  (Casado, Santillo and Johnston, 2018) |  |  |  |
| Bromoxynil | <0.02 – 0.1 | 700 | England: Anglian | (Croll, 1991) |  | 7079 |  |
| Carbendazim | 0.0006 – 0.0016 | 4 | England: Southwest | (Casado, Santillo and Johnston, 2018) |  | 6170 |  |
| Carbaryl | 0.01 – 0.12 | 24 | England: Northeast | (Long *et al.*, 1998) |  | 4499 |  |
| Chlorpyrifos | 0.2 – 2500 | 27 | England: Anglian | (Raven and George, 1989) | Growth: 3  (Ischnura elegans; 6 days^1^)  Development: 7.5E-03  (Daphnia magna; 72 hrs^1^)  Reproduction: 0.021  (Ceriodaphnia dubia; 7 days^5^)  Mortality: 0.02  (Paratya australiensis; 48 hrs^6^) | 5843 | Growth: 0.03%  Development: 2%  Reproduction: 0.5%  Mortality: 0.9% |
| Chlortoluron | <0.05 – 2.6 | 1012 | England: Anglian,  Southeast | (Croll, 1991) |  | 11975 |  |
|  |  |  |  | (Neal *et al.*, 2000) |  |  |  |
| cis-Permethrin | 0.02 – 0.37 | 24 | England: Northeast | (Long *et al.*, 1998) |  | 7370 |  |
| Clomazone | 0.002 | 4 | England: Southwest | (Casado, Santillo and Johnston, 2018) |  |  |  |
| Clothianidin | 0 – 0.39 | 43 | Scotland  Northern Ireland  Wales  England: Northeast, Northwest, Southeast, Thames, Midlands, Anglian | (Buglife, 2017)  (Casado, Santillo and Johnston, 2018)  (Egli *et al.*, 2023) |  |  |  |
| Cyanazine | 0.01 – 0.51 | 24 | England:  Northeast | (Long *et al.*, 1998) |  | 3972 |  |
| Cypermethrin** | 0.000005 - 0.001296 | 280 | England: Northeast, Northwest, Southeast, Thames, Midlands, Anglian | (Environment Agency, 2019) | Reproduction: 2E-06  (Daphnia magna; 21 days^1^)  Growth: 950  (Alona guttata; 7 days^1^)  Mortality: 0.002  (Palaemonetes argentinus; 21 days^1^) | 9939 | Reproduction: 100%  Growth: 0.1%  Mortality: 36% |
| Cyromazine | 0.022 – 0.038 | 5 | England:  Thames | (Egli *et al.*, 2023) |  |  |  |
| DEET (N,N-Diethyl-m-toluamide) | 19 | 11595 |  | (Spurgeon *et al.*, 2021) |  |  |  |
| Deltamethrin | 0.05 | 24 | England:  Northeast | (Long *et al.*, 1998) | Growth: 0.005  (Ceriodaphnia dubia; 8 days^1^)  Development: 0.02  (Daphnia magna; 21 days^1^)  Reproduction: 5.4E-03  (Ceriodaphnia dubia; 8 days^5^)  Mortality: 6E-06  (Chironomus riparius; 28 days^1^) | 2384 | Growth: 0.4%  Development: 0.3%  Reproduction: 0.4%  Mortality: 100% |
| Desmetryn | 0.03 – 0.73 | 24 | England:  Northeast | (Long *et al.*, 1998) |  | 6198 |  |
| Diazinon | 0.0061 – 0.86 | 724 | England:  Northeast,  Anglian,  Southwest | (Long *et al.*, 1998) | Growth: 0.53  (Daphnia magna; 21 days^3^)  Reproduction: 2E-04  (Daphnia magna; 21 days^3^)  Mortality: 3.5E-04  (Daphnia magna; 21 days^2^) | 15111 | Growth: 0.03%  Reproduction: 97%  Mortality: 95% |
|  |  |  |  | (Croll, 1991) |  |  |  |
|  |  |  |  | (Proctor *et al.*, 2019) |  |  |  |
| Dicamba | <0.1 – 0.3 | 700 | England: Anglian | (Croll, 1991) |  | 12333 |  |
| Dichloroprop | <0.1 – 0.5 | 700 | England: Anglian | (Croll, 1991) |  | 12544 |  |
| Dichlorvos | 0.001 – 0.002 | 9828 | England:  Anglian, Southwest, Midlands, Southeast, Thames, Northeast, Northwest  Wales | (Comber, Mistry and Sturdy, 2012) |  | 17119 |  |
| Dimethenamid | 0.0115 – 230 | 1095 | England: Southwest | (Spurgeon *et al.*, 2021) |  |  |  |
|  |  |  |  | (Casado, Santillo and Johnston, 2018) |  |  |  |
| Dimethoate | 0.01 – 0.94 | 724 | England: Northeast, Anglian | (Long *et al.*, 1998)  (Croll, 1991) |  | 8809 |  |
| Diuron | <0.04 – 0.56 | 2834 | England:  Southeast | (Spurgeon *et al.*, 2021) |  | 11981 |  |
|  |  |  |  | (Neal *et al.*, 2000) |  |  |  |
| Epoxiconazole | 0.003- 770 | 4878 | England:  Southwest, Anglian, Midlands | (Environment Agency, 2005) |  |  |  |
|  |  |  |  | (Casado, Santillo and Johnston, 2018) |  |  |  |
|  |  |  |  | (Spurgeon *et al.*, 2021) |  |  |  |
| Ethofumesate | 0.52 | 1 | England:  Southwest, Anglian, Midlands | (Environment Agency, 2005) |  | 5360 |  |
| Fenuron | 0.0025 – 0.0039 | 4 | England:  Southwest | (Casado, Santillo and Johnston, 2018) |  | 10432 |  |
| Fenitrothion | 0.001 – 0.22 | 9211 | England:  Anglian, Southwest, Midlands, Southeast, Thames, Northeast, Northwest  Wales | (Long *et al.*, 1998)  (Comber, Mistry and Sturdy, 2012) | Growth: 0.011  (Daphnia magna; 21 days^1^)  Reproduction:  0.011  (Daphnia magna; 21 days^1^) | 14541 | Growth: 8%  Reproduction: 8% |
| Fenvalerate | 0.08 – 0.14 | 24 | England:  Northeast | (Long *et al.*, 1998) | Growth: 0.01  (Limnephilus lunatus; 243.52 days^1^)  Development: 0.096  (Limnephilus lunatus; 244 days^1^)  Reproduction: 0.01  (Cloeon dipterum; 29 days^1^)  Mortality: 0.0039 (Ceriodaphnia quadrangular; 0.0417 days^4^) |  |  |
| Fipronil | 0.98 | 2603 | England | (Spurgeon *et al.*, 2021) | Morality: 0.257  (Hexagenia sp.; 96 hrs^2^) |  |  |
| Flusilazole | 0.07 | 1 | England:  Southwest, Anglian, Midlands | (Environment Agency, 2005) |  | 173 |  |
| Flufenacet | 0.024 - 98 | 2293 | England:  Southwest | (Spurgeon *et al.*, 2021)  (Proctor *et al.*, 2019) |  |  |  |
| Flutriafol | 0.009 – 0.05 | 25 | England:  Northeast,  Southwest, Anglian, Midlands | (Environment Agency, 2005) |  | 94 |  |
|  |  |  |  | (Long *et al.*, 1998) |  |  |  |
| Fluxapyroxad | 190 | 196 | England | (Spurgeon *et al.*, 2021) |  |  |  |
| Griseofulvin | 0.0012 – 0.0022 | 4 | England:  Southwest | (Casado, Santillo and Johnston, 2018) |  |  |  |
| Glyphosate | 0.24 - 3.34 | 3 | England:  Southwest, Anglian, Midlands | (Environment Agency, 2005) |  | 2848 |  |
| Imidacloprid | 0 – 0.36 | 1514 | Scotland  Northern Ireland  Wales  England: Northeast, Northwest, Southeast, Thames, Midlands, Anglian | (Buglife, 2017)  (Perkins *et al.*, 2021)  (Egli *et al.*, 2023)  (Casado, Santillo and Johnston, 2018)  (Proctor *et al.*, 2019) | Reproduction:  0.07  (Gammarus fossarum; 48 hrs^1^)  Mortality:  1.09E-04  (Cloeon sp.; 96 hrs^4^) |  |  |
| Ioxynil | <0.04 – 0.1 | 700 | England: Anglian | (Croll, 1991) |  | 11426 |  |
| Isoproturon | <0.04 – 11.5 | 1027 | England:  Southeast, Anglian, Southwest, Midlands | (Environment Agency, 2005)  (Croll, 1991)  (Neal *et al.*, 2000) |  | 13555 |  |
| Lindane | 0.01 – 3700 | 1004 | England:  Northeast,  Anglian,  Southeast | (Long *et al.*, 1998)  (Croll, 1991)  (Dowson *et al.*, 1996) | Growth: 6.11  (Gammarus pulex; 14 days^1^)  Reproduction:  10.5  (Ceriodaphnia dubia; 7 days^1^)  Mortality: 0.8  (Chironomus riparius; 10 days^2^) | 16854 | Growth: 0.006%  Reproduction: 0.006%  Mortality: 0.04% |
| Linuron | 0.09 - <0.2 | 724 | England: Northeast,  Anglian | (Long *et al.*, 1998)  (Croll, 1991) |  | 14208 |  |
| Malathion | 0.001 – 0.11 | 9517 | England:  Anglian, Southwest, Midlands, Southeast, Thames, Northeast, Northwest  Wales | (Long *et al.*, 1998)  (Comber, Mistry and Sturdy, 2012) | Growth:  0.1  (Daphnia magna; 21 days^1^)  Reproduction: 0.1  (Daphnia magna; 21 days^1^)  Mortality: 0.25  (Daphnia magna; 21 days^1^) | 14711 | Growth: 0.06%  Reproduction: 0.06%  Mortality: 0.03% |
| MCPB | <0.05 | 312 | England:  Southeast | (Neal *et al.*, 2000) |  | 7534 |  |
| MCPA | <0.05 – 16.0 | 1016 | England: Anglian,  Southwest,  Southeast | (Croll, 1991)  (Casado, Santillo and Johnston, 2018)  (Neal *et al.*, 2000) |  | 15572 |  |
| Mecoprop | 0.0062 – 5.1 | 1012 | England: Anglian,  Southeast | (Croll, 1991)  (Zhang and Zhou, 2007)  (Neal *et al.*, 2000) |  | 19071 |  |
| Metamitron | 0.0033 | 4 | England: Southwest | (Casado, Santillo and Johnston, 2018) |  |  |  |
| Metaldehyde | 0.002 - 6.780 | 2755 | England:  Southeast,  Anglian, Northwest, Thames  Wales | (Kay and Grayson, 2014)  (Castle *et al.*, 2018)  (Balashova *et al.*, 2021)  (Castle *et al.*, 2019) |  | 1626 |  |
| Metazachlor | 0.0045 - 340 | 2038 | England:  Southwest, Anglian, Midlands | (Environment Agency, 2005)  (Spurgeon et al., 2021)  (Casado, Santillo and Johnston, 2018)  (Proctor *et al.*, 2019) |  | 4255 |  |
| Metolachlor | 0.0012 – 0.0044 | 4 | Southwest England | (Casado, Santillo and Johnston, 2018) |  |  |  |
| Nicosulfuron | 0.0072 | 4 | Southwest England | (Casado, Santillo and Johnston, 2018) |  |  |  |
| Oxadiazon | 0.0169 |  | England:  Southwest | (Proctor *et al.*, 2019) |  |  |  |
| Parathion | 0.01 – 0.05 | 24 | England: Northeast | (Long *et al.*, 1998) | Mortality: 3.1E-04  (Daphnia magna; 24 hrs^2^) | 11486 | Mortality: 100% |
| Pencycuron | 0.0027 | 4 | England: Southwest | (Casado, Santillo and Johnston, 2018) |  |  |  |
| Piperophos | 0.007 – 0.011 | 3 | England:  Thames | (Egli *et al.*, 2023) |  |  |  |
| Pirimicarb | 0.0028 | 4 | England: Southwest | (Casado, Santillo and Johnston, 2018) |  | 5838 |  |
| Pendimethalin | 0.02-0.76 | 13 | England:  Southwest, Anglian, Midlands | (Environment Agency, 2005) |  | 3802 |  |
| Piperonyl butoxide | 0.0028 | 24 | England: Northeast | (Burns *et al.*, 2017) |  |  |  |
| Phenmedipham | 3060 | 18 | England | (Spurgeon et al., 2021) |  | 10 |  |
| Prometryn | 0.0028 – 2.44 | 28 | England: Northeast,  Southwest | (Long *et al.*, 1998)  (Casado, Santillo and Johnston, 2018) |  | 6203 |  |
| Propanil | 0.01 | 24 | England: Northeast | (Long *et al.*, 1998) |  |  |  |
| Propamocarb | 0.005 – 0.018 | 21 | England:  Thames | (Egli *et al.*, 2023) |  |  |  |
| Propazine | <0.05 – 3.12 | 336 | England: Northeast,  Southeast | (Long *et al.*, 1998)  (Neal *et al.*, 2000) |  |  |  |
| Propiconazole | 0.082 | 2404 | England | (Spurgeon *et al.*, 2021) |  |  |  |
| Propyzamide | 0.0029 – 72 | 5940 | England: Southwest,  Anglian, Midlands | (Environment Agency, 2005)  (Spurgeon *et al.*, 2021)  (Casado, Santillo and Johnston, 2018)  (Croll, 1991) |  | 5600 |  |
| Simazine | 0.006 – 7.1 | 3843 | England: Northeast,  Southeast,  Anglian,  Thames, Midlands | (Neal *et al.*, 2000)  (Environment Agency, 2005)  (Long *et al.*, 1998)  (Croll, 1991)  (Egli *et al.*, 2023) |  | 17414 |  |
| Spiroxamine | 0.01 | 4 | England: Southwest | (Casado, Santillo and Johnston, 2018) |  |  |  |
| Tebuconazole | 0.0114 – 210 | 305 | England:  Southwest, Anglian, Midlands | (Environment Agency, 2005)  (Casado, Santillo and Johnston, 2018)  (Spurgeon et al., 2021) | Growth: 20  (Attheyella crassa; 21 days^1^)  Development: 192  (Daphnia longispina; 21 days^1^)  Reproduction: 25  (Daphnia magna; 21 days^1^)  Mortality: 13.5  (Daphnia galeata; 28 days^1^) |  |  |
| Terbuthylazine (TERBA) | 0.0062 – 118 | 100 | England: Southwest | (Spurgeon et al., 2021)  (Casado, Santillo and Johnston, 2018) |  |  |  |
|  |  |  |  |  |  |  |  |
| Terbutryn | 0.004 – 9.3 | 1339 | England: Northeast,  Southwest,  Thames, Anglian, Midlands | (Environment Agency, 2005)  (Long *et al.*, 1998)  (Spurgeon *et al.*, 2021)  (Casado, Santillo and Johnston, 2018)  (Egli *et al.*, 2023) |  | 8893 |  |
| Thiacloprid | 0 – 0.0005 | 23 | Scotland  Northern Ireland  Wales  England: Northeast, Northwest, Southeast, Thames, Midlands, Anglian | (Buglife, 2017) |  |  |  |
| Thiabendazole | 0.0005 | 4 | England: Southwest | (Casado, Santillo and Johnston, 2018) |  |  |  |
| Thiamethoxam | 0 – 0.06 | 23 | Scotland  Northern Ireland  Wales  England: Northeast, Northwest, Southeast, Thames, Midlands, Anglian | (Buglife, 2017) |  |  |  |
| trans-Permethrin | 0.01 – 0.06 | 24 | England: Northeast | (Long *et al.*, 1998) |  | 7009 |  |
| Triallate | 0.98 | 1158 | England | (Spurgeon *et al.*, 2021) |  | 254 |  |
| Tributyltin | <0.04 – 5200 | 180 | England: Southeast | (Dowson *et al.*, 1996) | Mortality: 72  (Brachionus calyciflorus; 24 hrs^2^) | 21048 | Morality: 0.19% |
| Trifluralin | 0.012 – 6.01 | 30 | England: Northeast,  Southwest, Anglian, Midlands | (Environment Agency, 2005) |  | 8501 |  |
|  |  |  |  | (Long *et al.*, 1998) |  |  |  |
| Triphenyltin | 0.001 – 0.074 | 6,089 | England:  Anglian, Southwest, Midlands, Southeast, Thames, Northeast, Northwest  Wales | (Comber, Mistry and Sturdy, 2012) |  |  |  |

*The regions for which chemical samples were taken in England by Spurgeon *et al.*, (2021) is not specified. Cypermethrin (**) concentrations were taken from freshwaters. Endpoints: LOEC = 1, LC50 = 2, EC50 = 3, EC20 = 4, EC25 = 5, LC01 = 6.

The frequency with which pesticides have exceeded lowest effect concentrations for British invertebrates is not specified in table S2, as it was not feasible to derive such information from the literature. The reported concentration data varied in format- such as a range (min – max), mean and max only, or a list of individual concentrations – making it very difficult to consolidate the information.


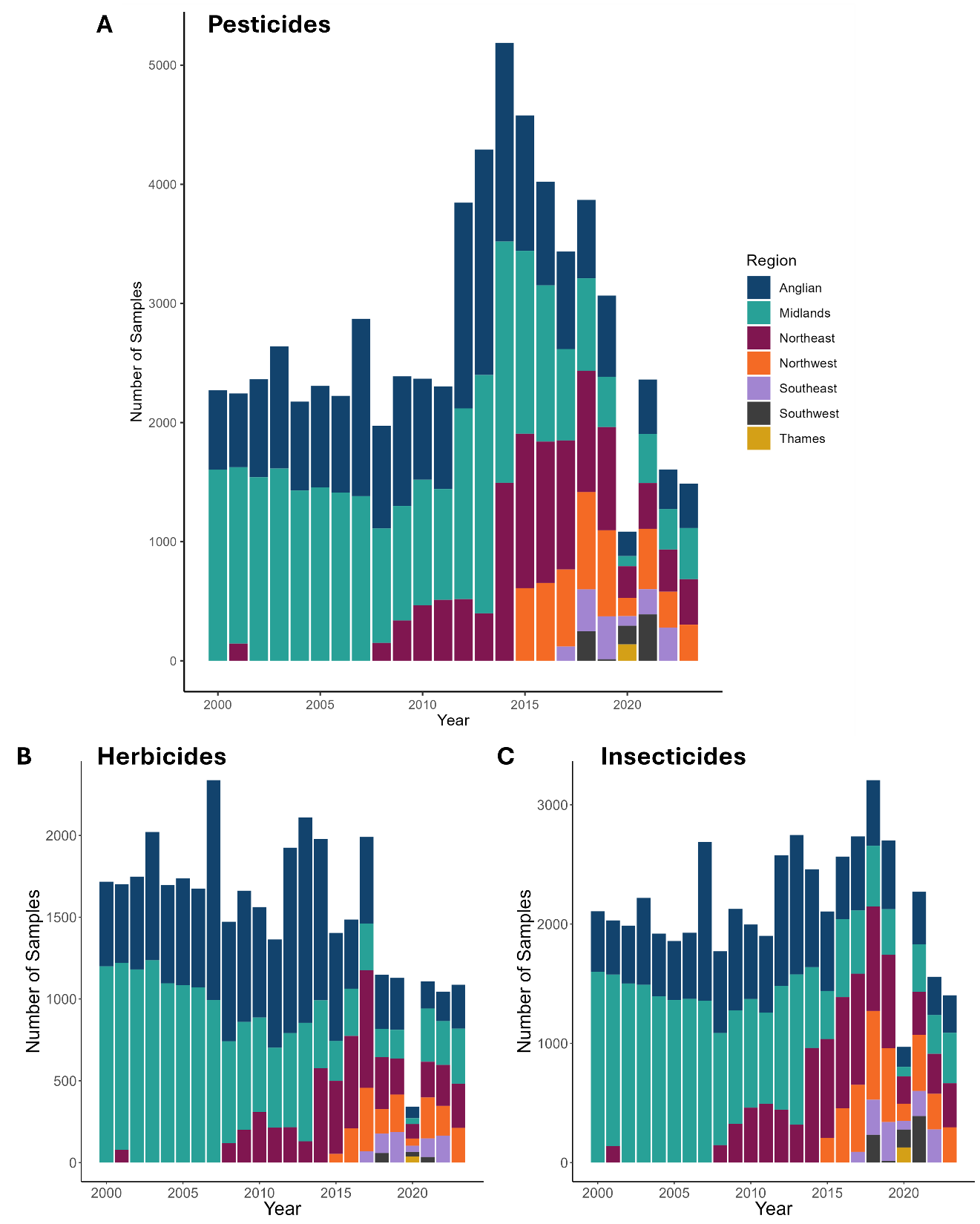


*205 pesticides were identified, some pesticides are classed as both insecticides and herbicides. *Number of entire samples.

**Figure S1: Number of samples collected and analysed for pesticide (also shown for the subclasses, insecticides and herbicides) in British rivers per year for the period between 2000-2023.**


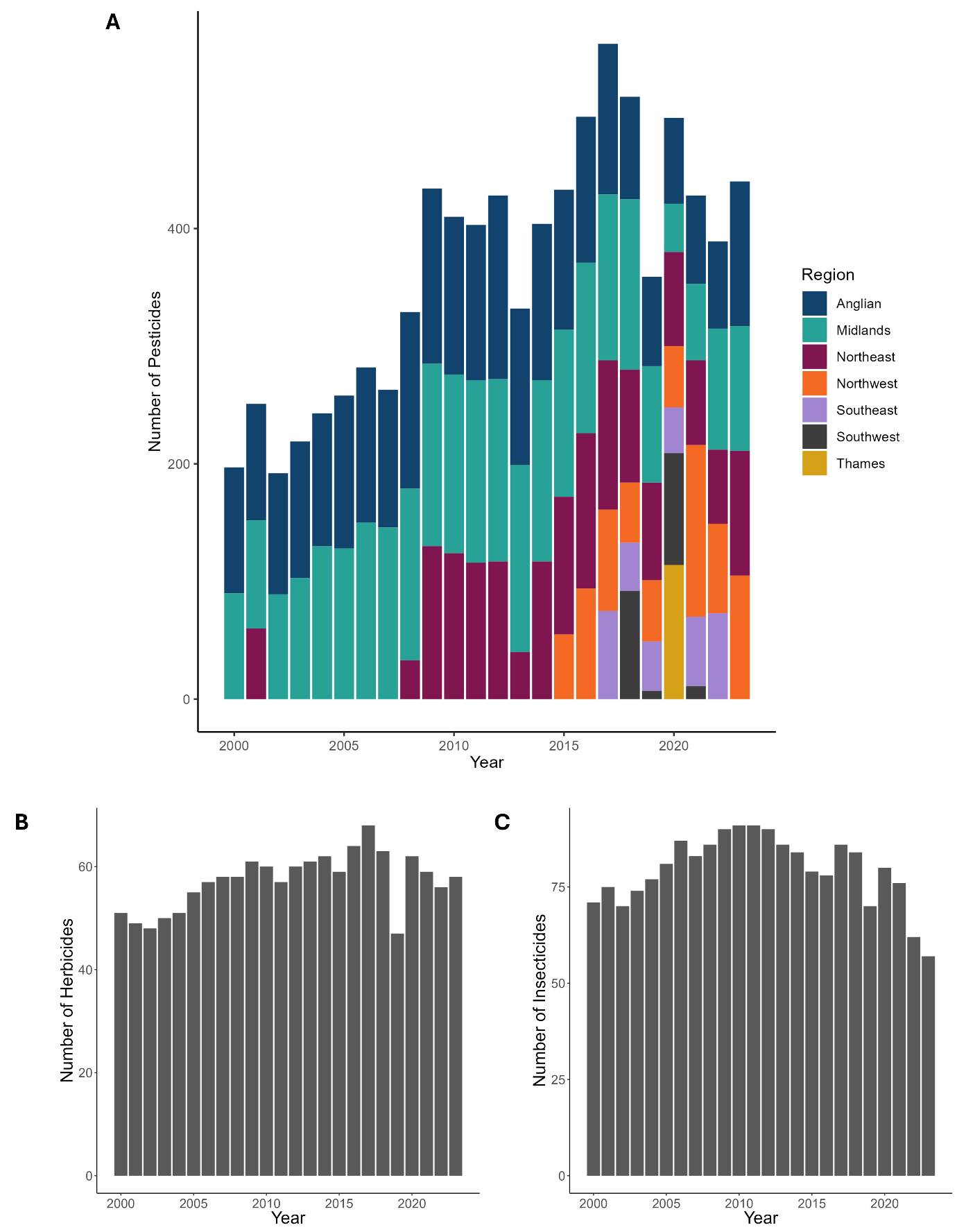


*205 pesticides were identified, some pesticides are classed as both insecticides and herbicides.

**Figure S2: Number of different pesticides (also shown for the subclasses, insecticides and herbicides) measured in British rivers per year for the period between 2000-2023.**


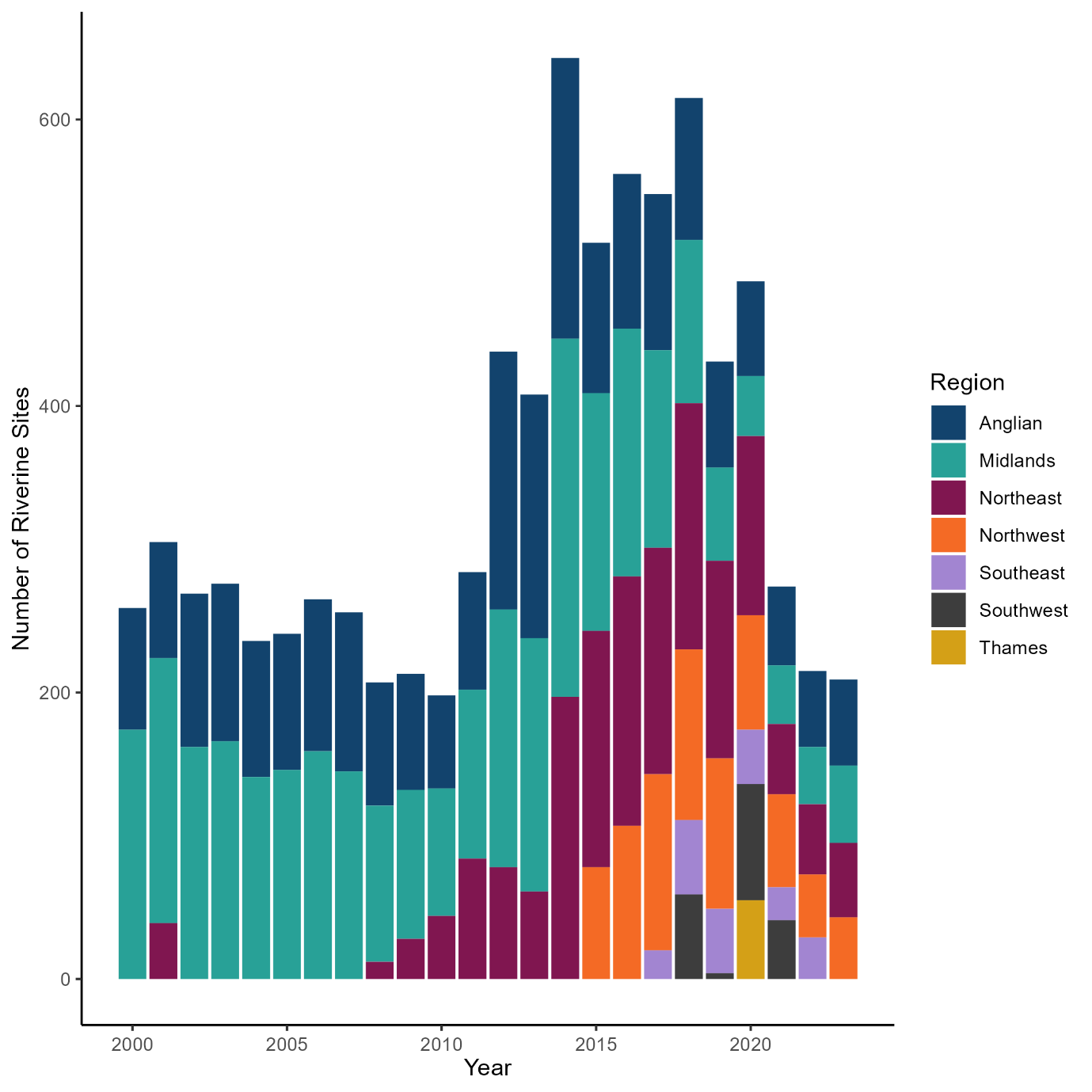


**Figure S3: Number of unique riverine sites sampled for pesticide residue measurements in British rivers per year for the period between 2000-2023.**


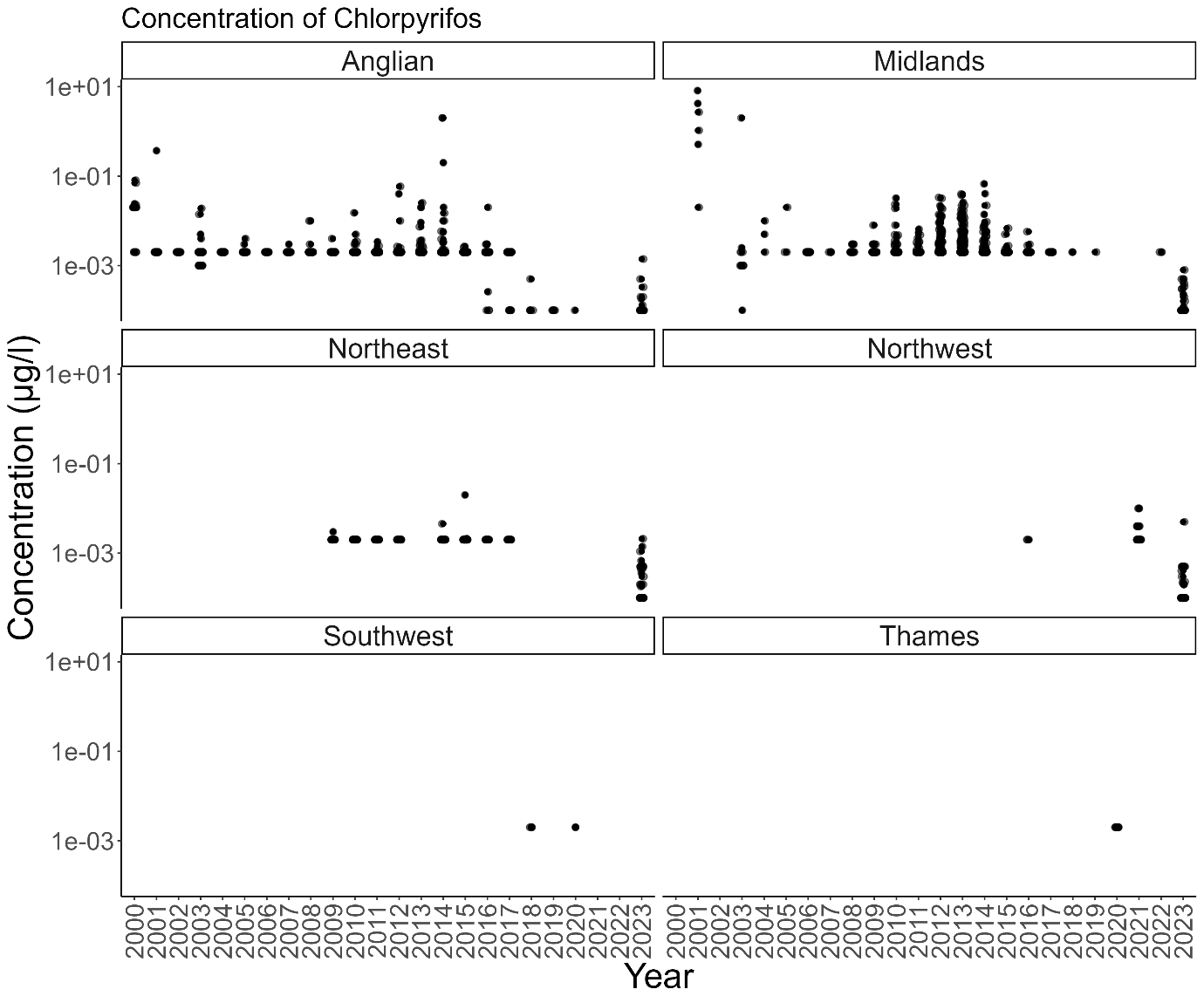


**Figure S4: Range of chlorpyrifos concentrations recorded in English rivers between years 2000 to 2023. Each black dot representents an individual datapoint (concentration) recorded from a site.**

**
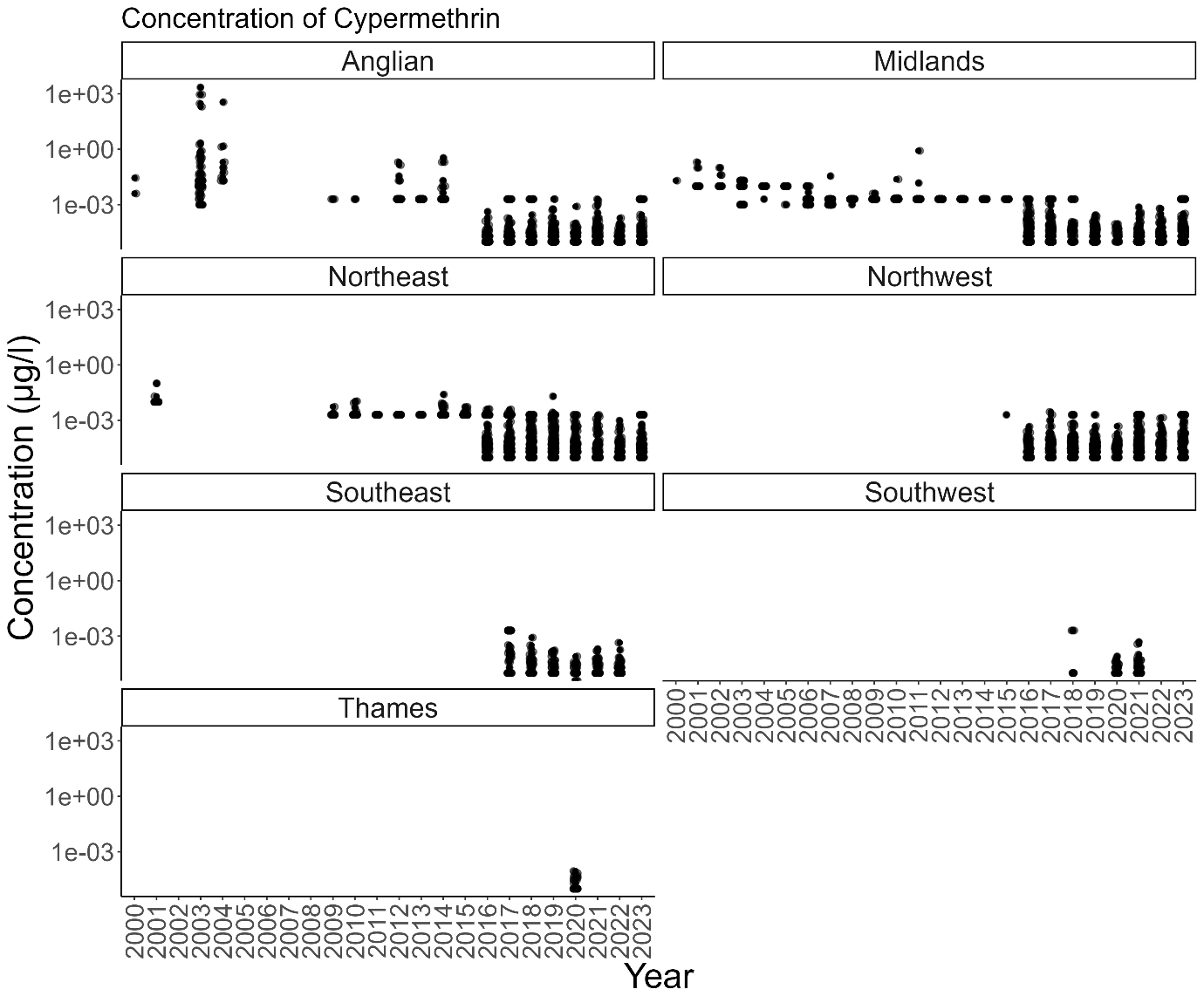
**

**Figure S5: Range of cypermethrin concentrations recorded in English rivers between years 2000 to 2023. Each black dot representents an individual datapoint (concentration) recorded from a site.**

**
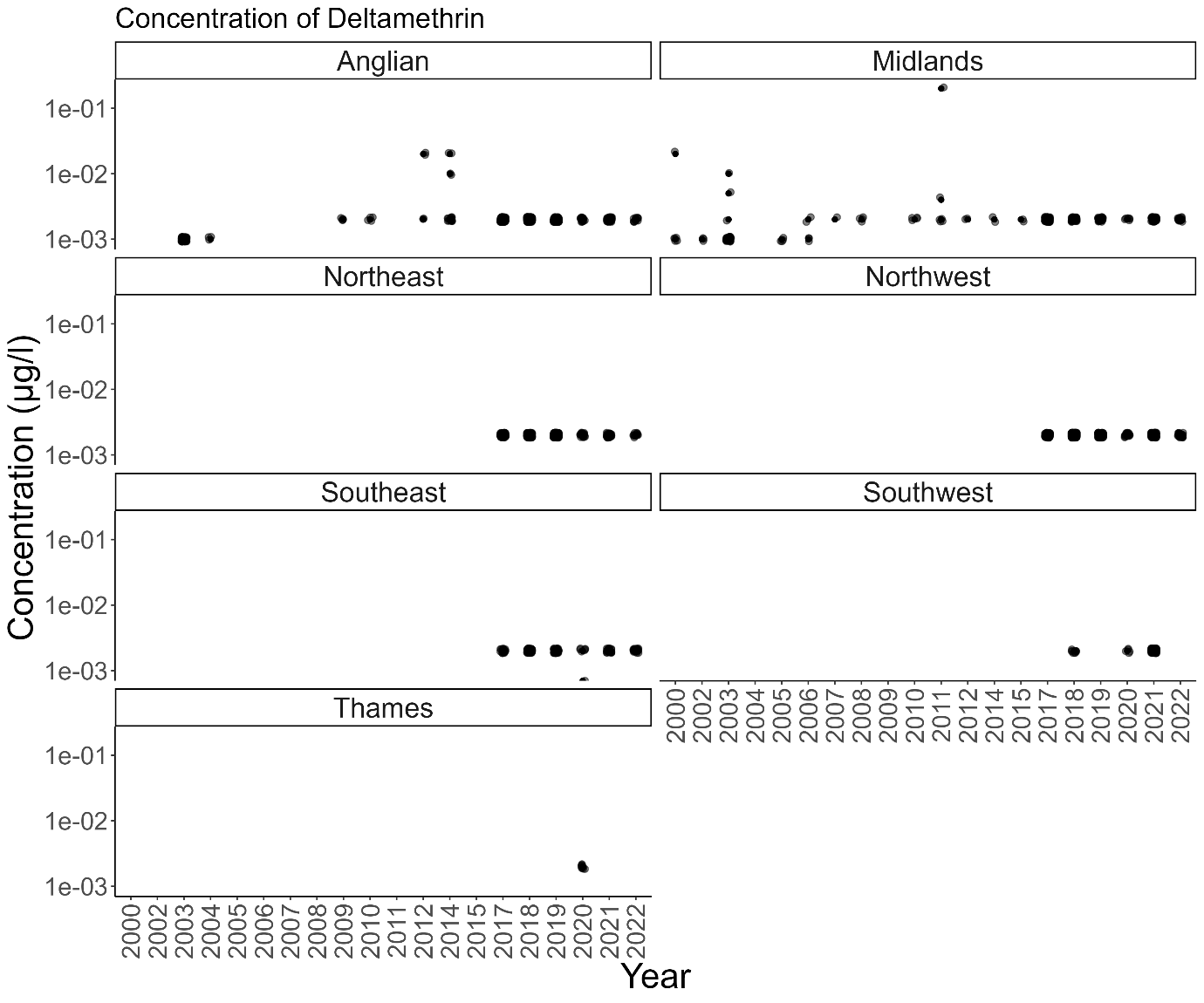
**

**Figure S6: Range of deltamethrin concentrations recorded in English rivers between years 2000 to 2023. Each black dot representents an individual datapoint (concentration) recorded from a site.**

**
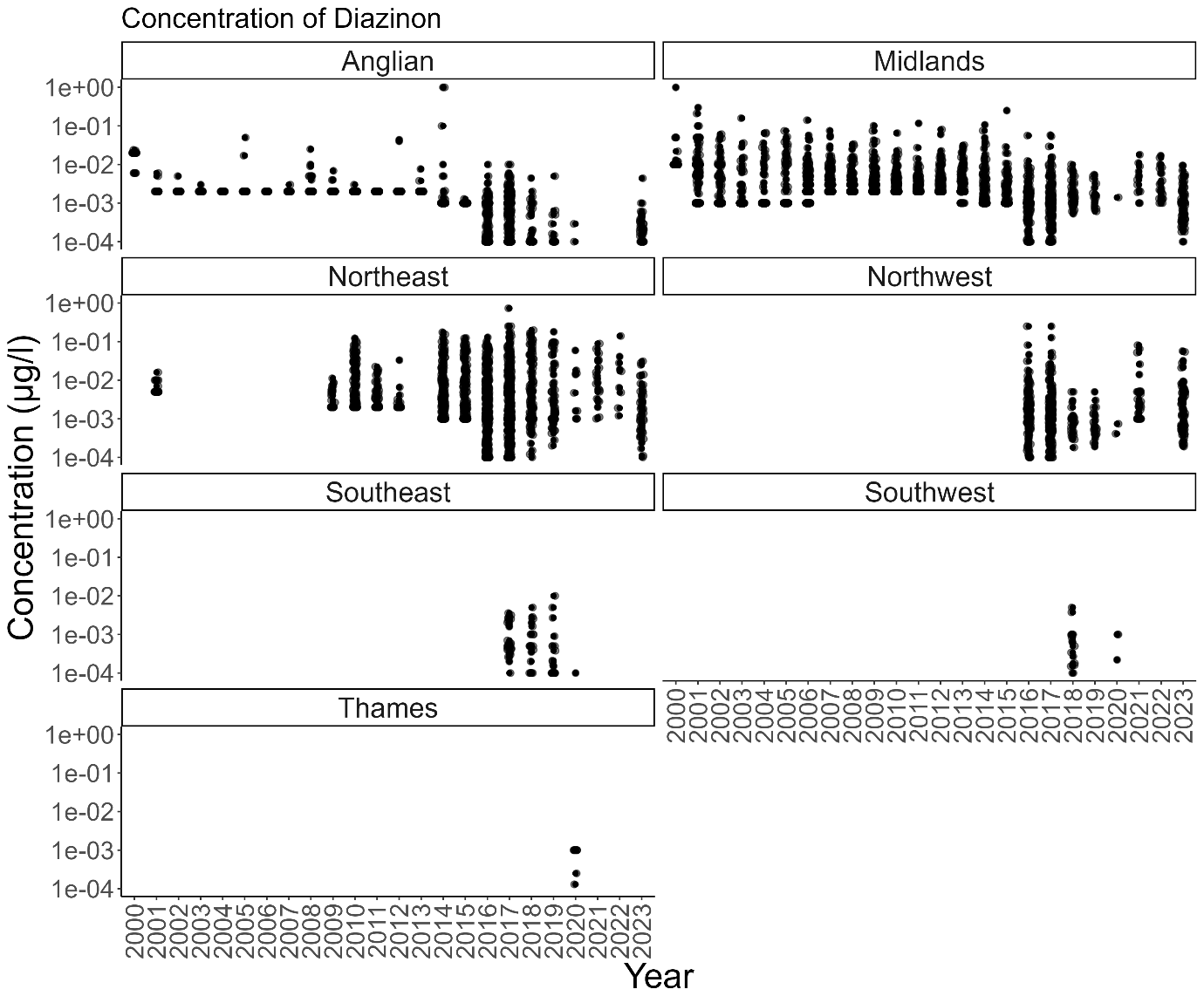
**

**Figure S7: Range of diazinon concentrations recorded in English rivers between years 2000 to 2023. Each black dot representents an individual datapoint (concentration) recorded from a site.**

**
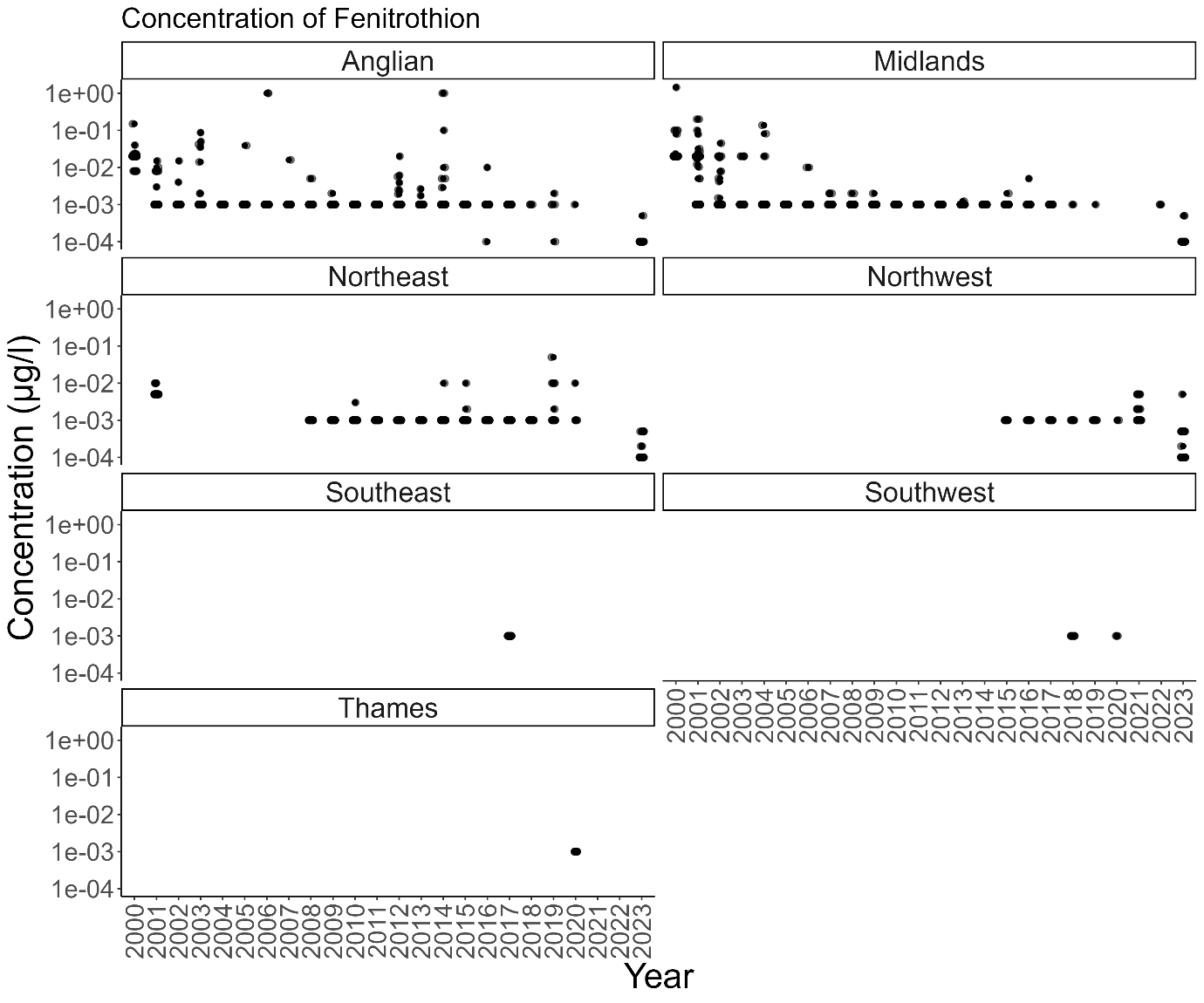
**

**Figure S8: Range of fenitrothion concentrations recorded in English rivers between years 2000 to 2023. Each black dot representents an individual datapoint (concentration) recorded from a site.**

**
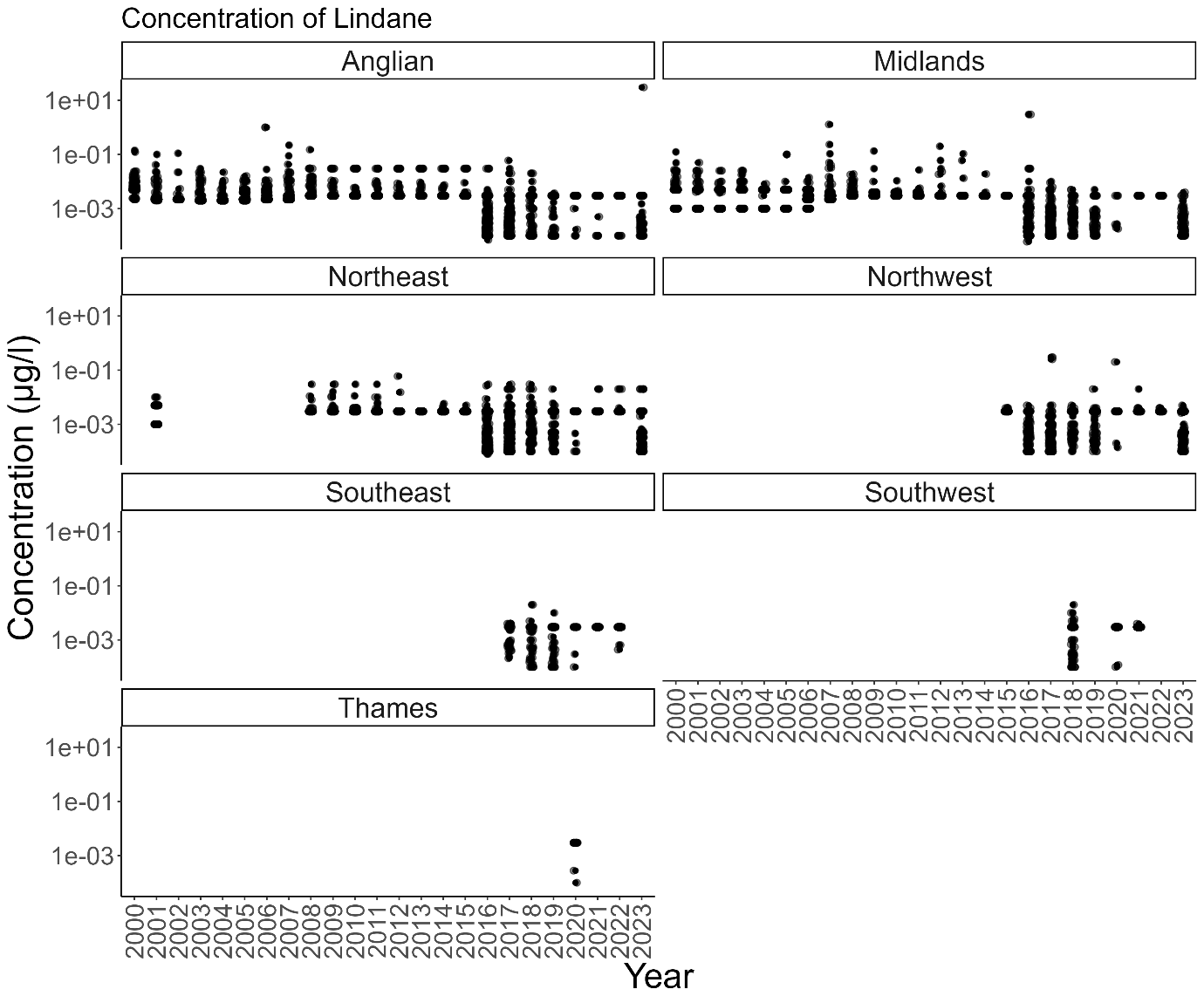
**

**Figure S9: Range of lindane concentrations recorded in English rivers between years 2000 to 2023. Each black dot representents an individual datapoint (concentration) recorded from a site.**

**
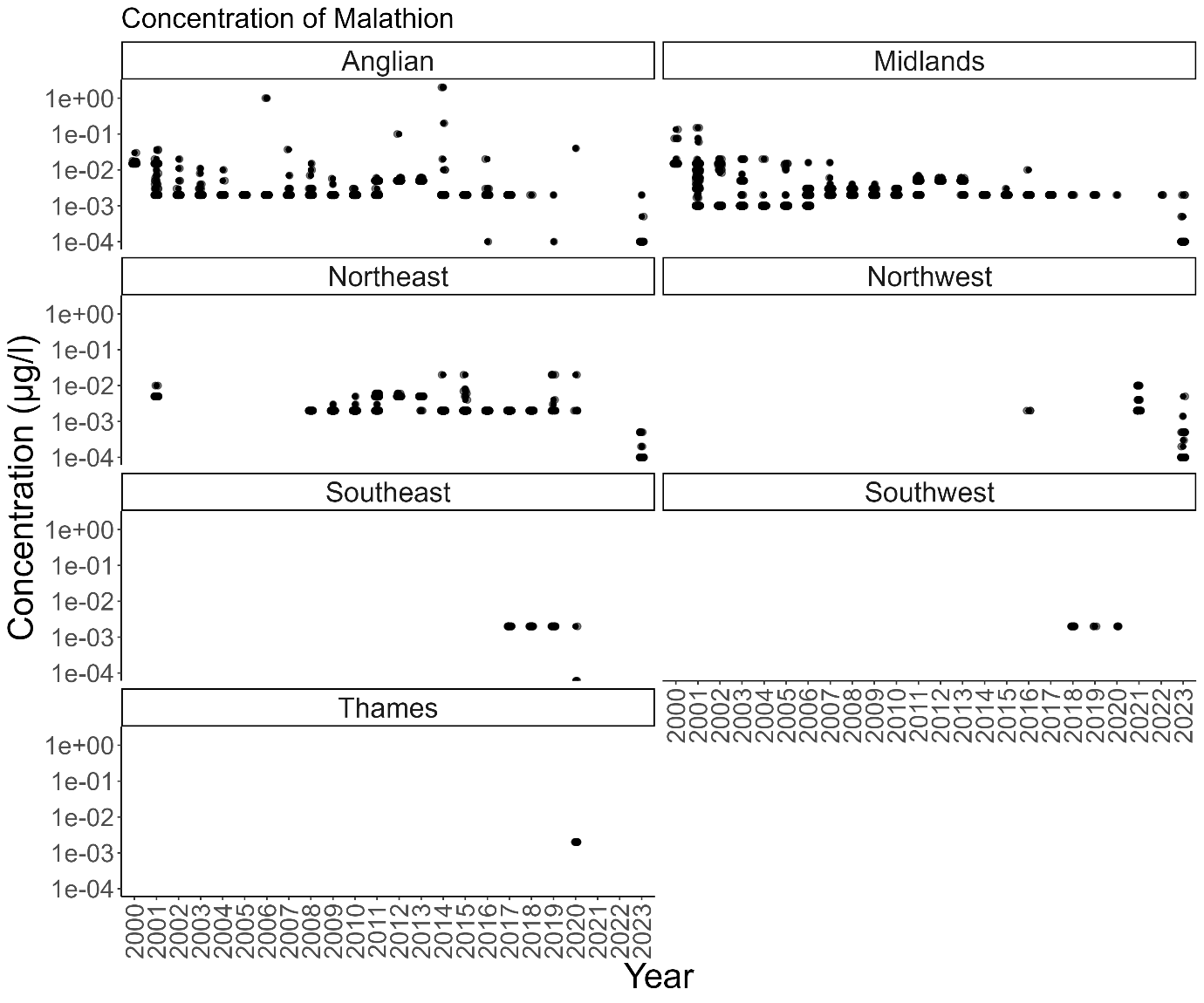
**

**Figure S10: Range of malathion concentrations recorded in English rivers between years 2000 to 2023. Each black dot representents an individual datapoint (concentration) recorded from a site.**

**
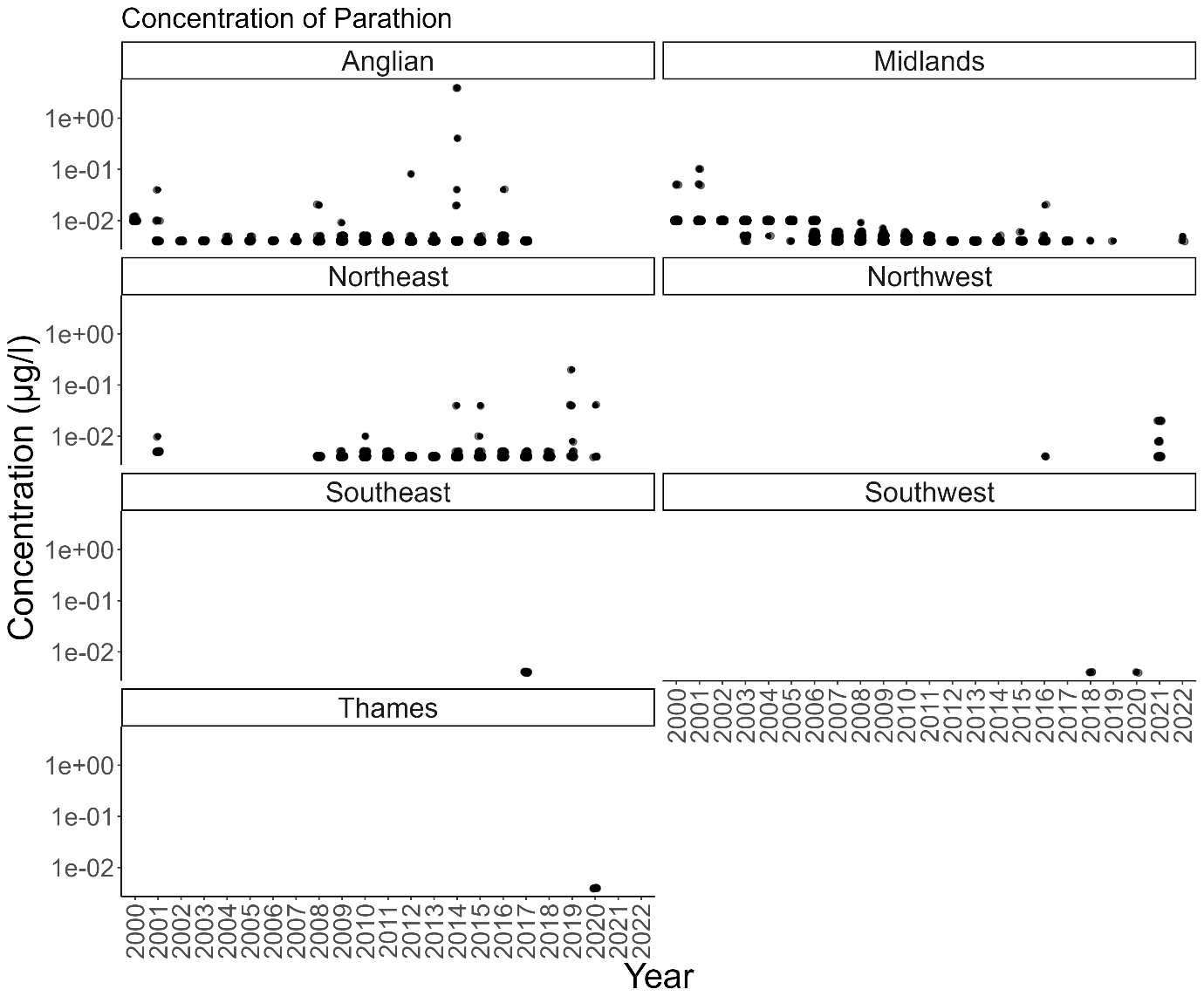
**

**Figure S11: Range of parathion concentrations recorded in English rivers between years 2000 to 2023. Each black dot representents an individual datapoint (concentration) recorded from a site.**

**
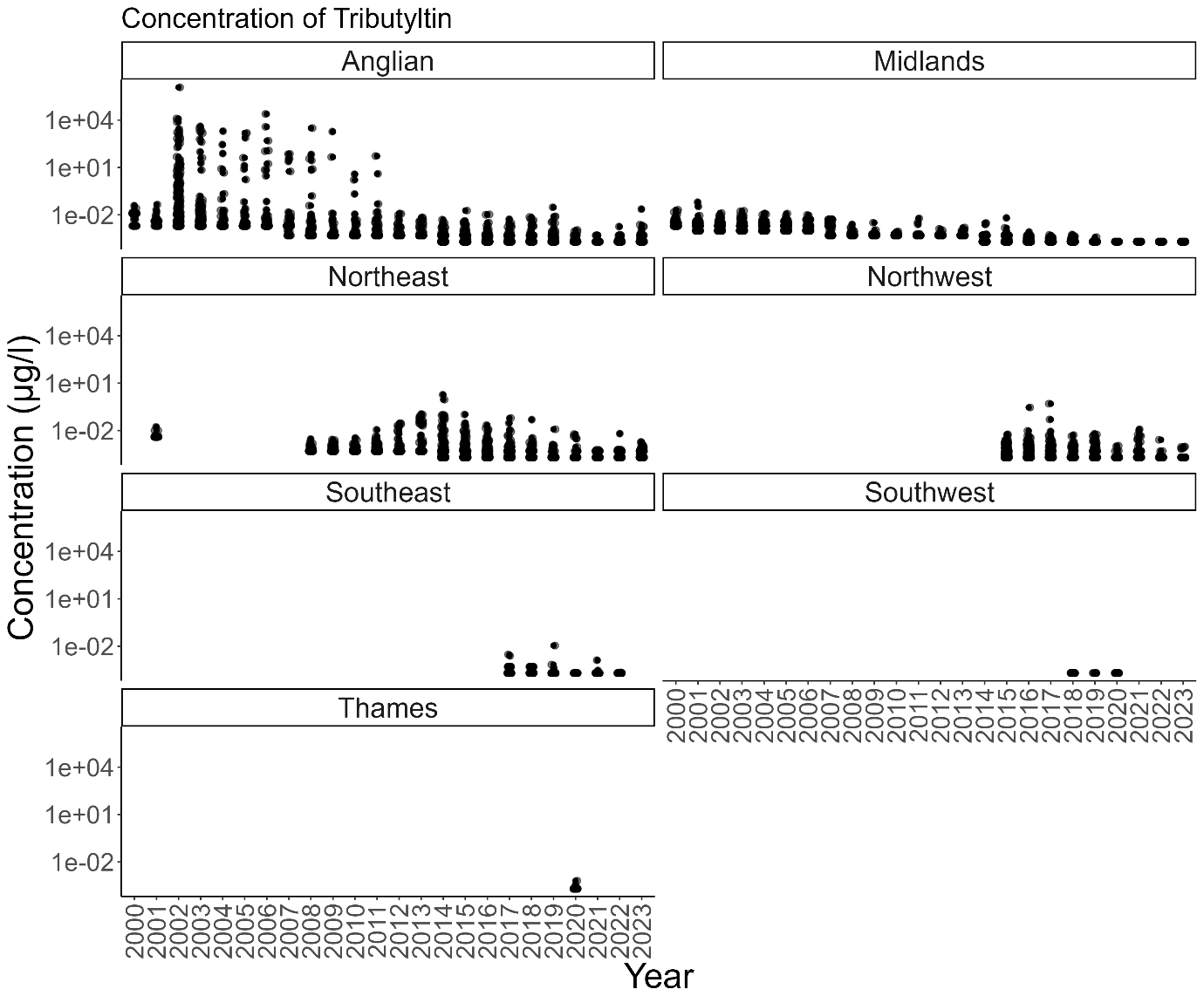
**

**Figure S12: Range of tributyltin concentrations recorded in English rivers since 2000 to 2023.**
